# Patterns of Anthelmintic Resistance in Gastrointestinal Nematodes of Small Ruminants: A Systematic Review and Meta-Analysis

**DOI:** 10.3390/vetsci13101077

**Published:** 2026-10-12

**Authors:** Songezo Mavundela, William Diymba Dzemo, Oriel Thekisoe

**Affiliations:** 1 Department of Biological and Environmental Sciences, Walter Sisulu University, Private Bag X1, Mthatha 5117, South Africa; 216011388@mywsu.ac.za; 2 Unit for Environmental Sciences and Management, North-West University, Potchefstroom 2531, South Africa; oriel.thekisoe@nwu.ac.za

**Keywords:** benzimidazoles, macrocyclic lactones, levamisole, *Haemonchus* spp., meta-regression, resistance, small ruminants

## Abstract

Anthelmintic resistance (AHR) in gastrointestinal nematodes (GINs) threatens sustainable parasite control in small ruminants worldwide. This study estimated the global prevalence of AHR and its associated factors through a PRISMA-compliant systematic review, meta-analysis, and meta-regression registered with PROSPERO. Studies published between 1996 and March 2026 were retrieved from PubMed, Web of Science, ScienceDirect, and Google Scholar. Random-effects meta-analysis estimated the pooled prevalence of reported AHR, while subgroup and multivariable meta-regression analyses identified study-level factors associated with the prevalence of reported resistance. A total of 4809 GIN populations from small ruminant farms across 44 countries were included in the meta-analysis. The overall pooled prevalence of reported AHR was 64.6% (95% CI: 61.7–67.5), with considerable between-study heterogeneity (I^2^ = 77%). Benzimidazoles and macrocyclic lactones exhibited the highest prevalence of reported resistance (69.7% and 68.6%, respectively), whereas resistance to imidazothiazoles was significantly lower than that of benzimidazoles (OR = 0.66, *p* < 0.05). Although *Haemonchus* spp. was the most frequently reported resistant genus, resistance prevalence did not differ significantly among the major GIN genera. Molecular techniques were associated with significantly higher odds of detecting resistance than the faecal egg count reduction test (OR = 4.04, *p* < 0.05). Geographically, North America showed significantly higher resistance than Africa (OR = 1.93, *p* < 0.05), whereas Oceania showed significantly lower odds (OR = 0.27, *p* < 0.0001). The odds of reported AHR increased by approximately 9% per study year (OR = 1.09, *p* < 0.0001). These findings highlight the urgent need for evidence-based parasite control and standardized resistance surveillance to slow the global spread of GIN in small ruminants.

## Simple Summary

Gastrointestinal nematodes are important parasites of sheep and goats that can negatively affect animal health, productivity, and farm income. These parasites are commonly controlled with anthelmintic drugs; however, repeated use of these drugs creates selection pressure for resistant worms, making treatment increasingly difficult. Previous reviews of anthelmintic resistance (AHR) have mainly focused on individual host species, risk factors, or specific geographic regions. Consequently, comprehensive global evidence on the magnitude of AHR and the effects of study-level factors on reported resistance prevalence remains limited. This study estimated the global prevalence of AHR and identified study-level factors associated with resistance using a systematic review, meta-analysis, and meta-regression. The findings indicate that AHR in GINs of small ruminants is widespread globally and has increased over time, with pharmacological, methodological, and geographical factors contributing to variation in reported resistance. The increasing burden of AHR highlights the limitations of relying on anthelmintic drugs alone for sustainable parasite control and supports the need to move beyond conventional drug-based control towards integrated parasite management.

## 1. Introduction

Sheep and goat farming contributes substantially to food security and rural livelihoods worldwide by providing meat, milk, wool, hides, and income, while enhancing household financial security and social status [1]. However, small ruminant production is constrained by several factors, including limited veterinary services, poor husbandry practices, and infectious diseases such as parasitic infections [2]. Among parasitic diseases, gastrointestinal nematode (GIN) infections are among the most important constraints on productivity worldwide [1,2]. The predominant GINs of the family Trichostrongylidae, particularly *Haemonchus* spp., *Trichostrongylus* spp., and *Teladorsagia* spp., cause substantial production losses in sheep and goats [3,4,5]. In sheep, these infections have been associated with reductions of approximately 15% in weight gain, 22% in milk yield, and 10% in wool production, as reported in a systematic review and meta-analysis [6]. Severe infections also impair meat production and reproductive performance and increase the risk of anaemia, morbidity, and mortality [7].

Control of GIN infections relies predominantly on anthelmintic drugs, with benzimidazoles, imidazothiazoles, macrocyclic lactones, and salicylanilides representing the principal chemical classes used worldwide [2]. Combination formulations containing two or more of these drug classes are also widely used to broaden the spectrum of activity and improve treatment efficacy [2,8]. Despite their widespread use, GIN infections and their control continue to impose considerable economic burdens on small-ruminant production. Annual losses associated with helminth infections have been estimated at approximately USD 222 million in Australia, USD 45 million in South Africa, and USD 42 million in Uruguay, while annual expenditure on anthelmintic treatment against *Haemonchus* spp. in India has been estimated at approximately USD 103 million [9,10].

The continued dependence on anthelmintics, together with decades of frequent and often indiscriminate use, has increased selection pressure on parasite populations, accelerating the development of anthelmintic resistance (AHR) [1,11]. Anthelmintic resistance is defined as a heritable reduction in the susceptibility of a parasite population that enables parasites to survive drug concentrations that would normally be effective [12]. Resistance has been widely reported across different geographical regions, including Africa, Asia, Europe, Oceania, North America, and Latin America [13,14,15,16,17,18,19,20,21,22,23,24,25,26]. The widespread AHR threatens animal health, productivity, and the long-term sustainability of small ruminant production. This challenge is further compounded by the limited availability of new anthelmintic drug classes, owing to the high costs, lengthy development process, and low success rates associated with anthelmintic discovery [27,28,29]. This limitation further emphasises the need for integrated parasite control strategies that reduce reliance on anthelmintics and sustain the efficacy of existing drugs [30].

Diagnosis of AHR relies on in vivo, in vitro, and molecular methods [31,32]. The faecal egg count reduction test (FECRT) remains the most widely used standardised in vivo method for assessing anthelmintic efficacy in small ruminants [33]. Complementary in vitro assays, including the egg hatch and larval development tests, assess parasite responses to anthelmintics under laboratory conditions [31,34]. Molecular techniques detect genetic markers associated with resistance and may identify resistance-associated alleles at low frequencies [31,34,35,36]. These approaches assess different aspects of resistance and may therefore yield different estimates of resistance prevalence.

Previous systematic reviews have described AHR in sheep and goats globally [5], while meta-analyses have focused on individual host species and selected risk factors [11,37]. Several reviews have also been focused on specific geographical regions, including Europe, the Nordic-Baltic region, Ethiopia, and Africa [38,39,40,41]. Consequently, a comprehensive global synthesis of the prevalence of AHR and the effects of study-level factors, including anthelmintic drug class, GIN genus, host species, route of administration, diagnostic method, geographic region, and study year, on resistance prevalence is lacking. Therefore, this systematic review and meta-analysis aimed to estimate the global pooled prevalence of reported AHR in GINs of small ruminants and identify study-level factors associated with variation in resistance prevalence. By synthesising global evidence, this study provides a comprehensive understanding of the epidemiology of AHR and highlights important patterns in its global distribution.

## 2. Materials and Methods

### 2.1. Search Approach and Protocol Registration

The systematic review and meta-analysis followed the Preferred Reporting Items for Systematic Reviews and Meta-Analyses (PRISMA 2020) guidelines [42] (Table S1). The protocol was prospectively registered with PROSPERO (Registration ID: CRD420261334742) before the literature search, with no deviations from the registered protocol. Prospective registration was undertaken to enhance transparency, minimise the risk of unintended duplication, and provide a publicly accessible record of the review protocol and planned methods. A comprehensive literature search was performed to identify studies published from 1 January 1996 to 31 March 2026 that reported AHR in GIN populations on small-ruminant farms worldwide. The search strategy combined terms selected to cover the main concepts of the review, including host species, GINs, AHR, or anthelmintic efficacy, and major anthelmintic drug classes. Drug-class terms were used to provide broad coverage of different active compounds within each class. Keywords included: (“sheep” OR “goats” OR “small ruminants”) AND (“Anthelmintic resistance” OR “susceptibility” OR “efficacy”) AND (“Gastrointestinal nematodes” OR “endoparasites” OR “*Nematodirus* spp.” OR “*Cooperia* spp.” OR *“Haemonchus* spp.” OR “*Trichostrongylus* spp.” OR “*Teladorsagia* spp.”) AND (“anthelmintic drugs” OR “benzimidazoles” OR “macrocyclic lactones” OR “imidazothiazoles” OR “salicylanilides” OR “tetrahydropyrimidines” OR “amino-acetonitrile derivatives” OR “organophosphates”). The anthelmintic drug classes were classified according to the Consortium for Anthelmintic Resistance and Susceptibility (CARS) framework [43]. Searches were conducted across four electronic databases: PubMed, Web of Science, ScienceDirect, and Google Scholar (Table S2). In addition, forward and backward citation searches of eligible peer-reviewed articles were conducted to identify further relevant studies. All retrieved records were exported and managed using Microsoft Word 365 (Microsoft Corporation, Redmond, WA, USA). Duplicate records were removed prior to screening.

### 2.2. Inclusion and Exclusion Eligibility Criteria

Publications identified through the search process were screened based on the relevance of their titles and abstracts for inclusion in the systematic review and meta-analysis. Studies were excluded if they: (i) assessed AHR in dogs, cattle, or horses; (ii) investigated drivers of AHR in small ruminants without providing data on the prevalence of AHR; (iii) were preprint articles; (iv) examined the effects of anthelmintic drugs on small ruminants without providing data on AHR; (v) focused on the epidemiology of GINs or the mechanisms of AHR; (vi) were book chapters on anthelmintic drugs or AHR; and (vii) had inaccessible full texts after searching relevant databases and the university library. Full-text articles retained following the initial screening were accessed through the iYunivesithi Walter Sisulu library and subsequently evaluated for their relevance to the assessment of AHR in GIN of small ruminants.

Articles were included in the systematic review if they met the following criteria: (i) the article was available in full text and published in English; (ii) the study provided data on resistance to a specified anthelmintic drug class or compound and described the method used to diagnose resistance; (iii) the article reported the geographic location (country) where the study was conducted; and (iv) the study reported the presence of AHR in at least one small ruminant farm. For quantitative synthesis, studies were additionally required to provide sufficient data to calculate the proportion of resistant units and to report resistance data from at least two farms. The minimum two-farm criterion was applied to reduce reliance on single-farm observations when estimating pooled resistance prevalence and to ensure that quantitative estimates incorporated information from more than one farm- level population. Studies based on a single farm were therefore retained for qualitative synthesis but excluded from the meta-analysis.

A study was defined as an independent, eligible published research report, a farm as an individual livestock production unit, and a GIN population as a parasite population assessed for AHR. The analytical unit was the study-specific proportion of resistant units, calculated as the number of resistant units divided by the total number assessed.

For studies reporting multiple anthelmintic classes, class-specific resistance proportions were extracted as separate effect sizes using the corresponding resistant and total units. The GIN genus, host species, administration route, and detection method were extracted as study-level moderators. Two reviewers independently screened the retrieved records and assessed eligible studies for inclusion. Disagreements regarding study eligibility or data extraction were resolved through discussion and consensus between the reviewers. Inter-reviewer agreement was not formally quantified using Cohen’s κ.

### 2.3. Data Extraction and Analysis

Data extracted from the eligible full-text articles included the author(s), year of publication, continent/country, GIN species, small ruminant host species (sheep or goat), number of farm exhibiting AHR, number of farms assessing AHR, anthelmintic drug class, mode of drug administration, and bioassay techniques used for resistance detection (FECRT; LDT, larval development test; EHT, egg hatch test; micro-agar larval development test; Molecular (ASP, Allele-specific PCR, and DSP, Deep Amplicon Sequencing PCR) and the parameters used for the quantification of AHR. The bioassay techniques measure different manifestations of AHR, but the included studies classified their parasite population as resistant or non-resistant based on established method-specific criteria. These classifications were harmonised into a common binary study-level outcome (presence or absence of AHR at the study or population level).

Data from the included studies were summarised using descriptive statistics (counts and proportions) and grouped according to anthelmintic drug class, GIN species, bioassay technique, host species, and geography. Prior to meta-analysis, data from eligible studies were compiled in Microsoft Excel 365 (Microsoft Corporation, Redmond, WA, USA) and imported into Comprehensive Meta-Analysis (Version 4; Biostat, Inc., Englewood, NJ, USA). Multiple studies reported more than one anthelmintic drug class, GIN species, host species, mode of administration, and detection method. Each reported chemical class, GIN species, host species, and detection method was treated as a separate observation within the dataset.

A random-effects meta-analysis of proportions was conducted using event rates to estimate the pooled prevalence of reported AHR in GINs. This model assumes that the true effect sizes vary across studies and estimates an overall pooled prevalence as a weighted average of the study-specific effects. Between-study heterogeneity was assessed using Cochran’s Q test and quantified using Higgins’ I^2^ statistic, with values of <25%, 25–50%, 50–75%, and >75% indicating low, moderate, substantial, and considerable heterogeneity, respectively [44,45]. Heterogeneity measures were not reported for variables with fewer than five studies because the Cochran’s Q test has limited power to detect heterogeneity when the number of studies is small [45].

Subgroup meta-analyses were conducted by stratifying studies according to anthelmintic drug class, GIN species, host species, mode of administration, resistance detection method, and geographical region. Associations between resistance prevalence and study-level covariates were assessed using multilevel random-effects meta-regression, with logit-transformed proportions as the outcome. Predictor variables included anthelmintic drug class (reference: benzimidazoles), GIN species (reference: *Haemonchus* spp.), host species (reference: sheep), mode of administration (reference: oral), resistance detection method (reference: faecal egg count reduction test), geographical region (reference: Africa), and study year (continuous variable, scaled per 1-year increment). Reference categories were selected based on biological relevance, epidemiological rationale, interpretability, and adequate sample size. Regression coefficients (β) were exponentiated to obtain odds ratios. The proportion of between-study heterogeneity explained by the moderators was estimated using the R^2^ statistic [46,47]. Statistical significance was set at a threshold of *p* < 0.05 for all analyses. Publication bias was assessed by visual inspection of funnel plots.

## 3. Results

### 3.1. Characteristics of Studies on the Development of Anthelmintic Resistance in GIN Populations of Small Ruminants

**Figure 1:**
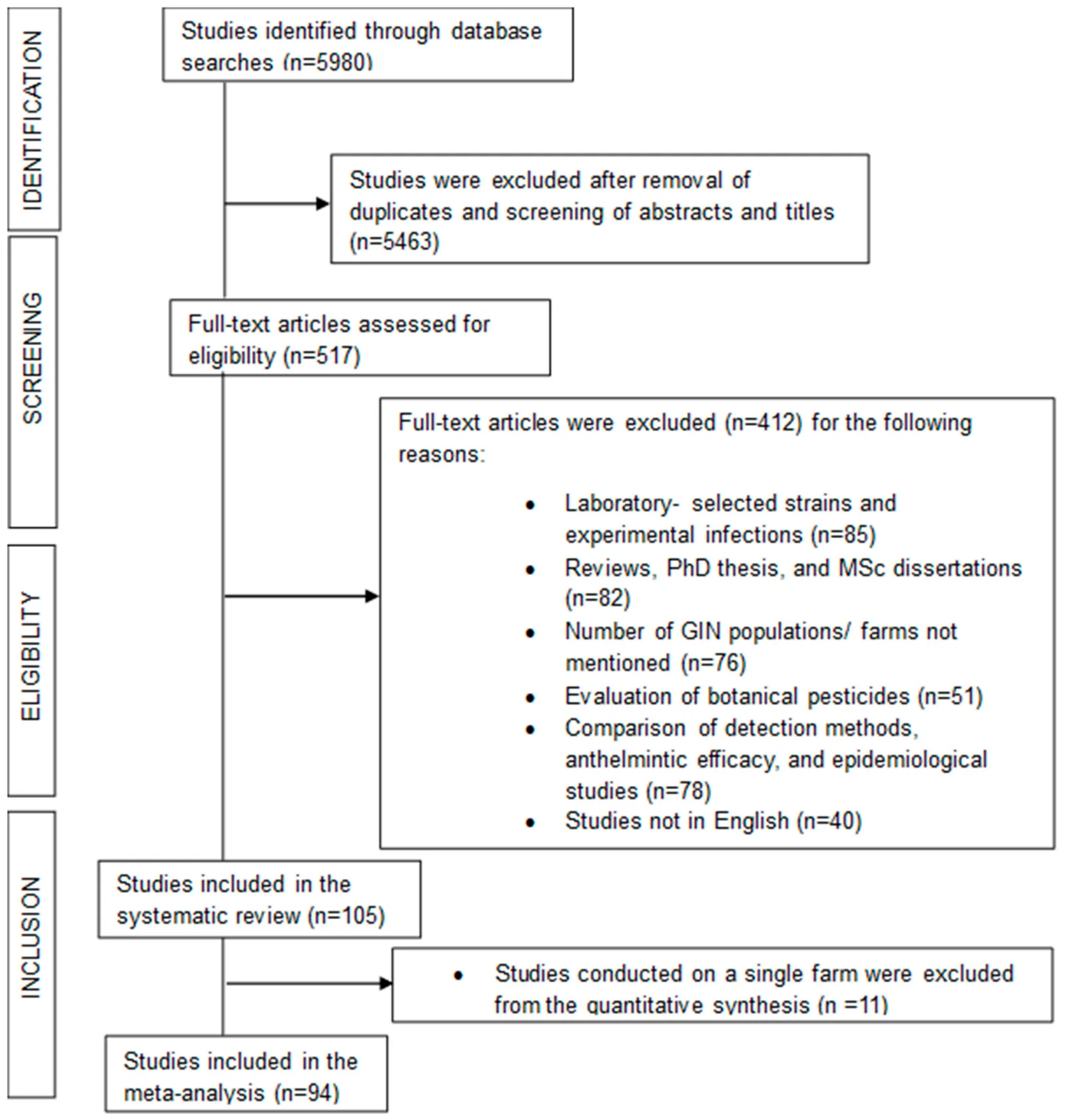
PRISMA flow diagram illustrating the selection of studies included in the systematic review and meta-analysis.

**Figure 2:**
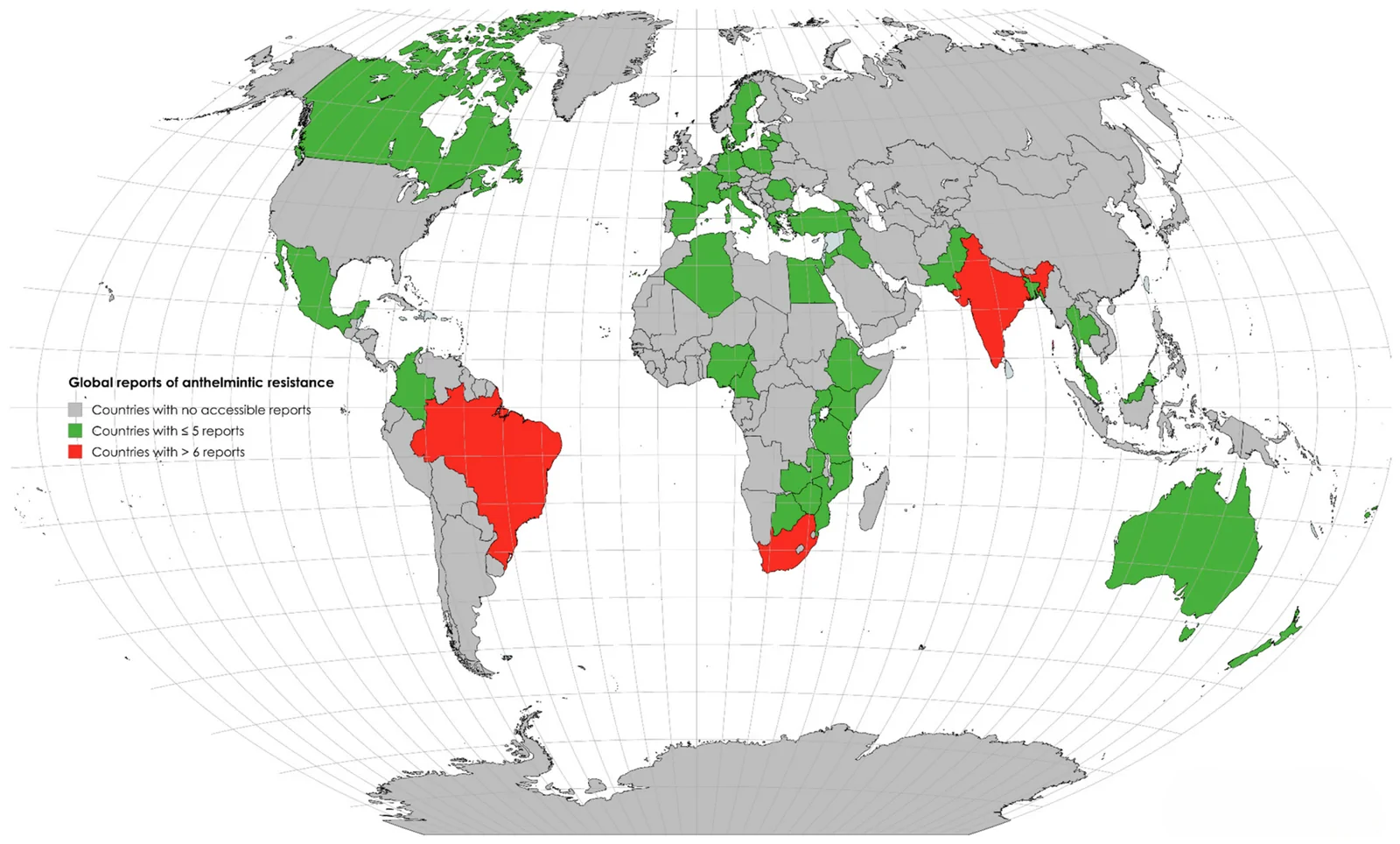
Global reports of anthelmintic resistance in small ruminants within the study period (1996–2026).

The literature search conducted using the iYunivesithi Walter Sisulu search engine identified 5980 studies from Google Scholar (*n* = 5290), ScienceDirect (*n* = 469), Web of Science (*n* = 174), and PubMed (*n* = 47). After title and abstract screening, 5463 publications were excluded as irrelevant or duplicates, leaving 517 studies for eligibility assessment. A total of 105 studies for the systematic review and 94 studies for the meta-analysis, published between 1996 and March 2026 met the inclusion criteria (Figure 1). Globally, studies on the development of AHR in GINs of small ruminants were mostly reported from Africa (*n* = 35, from 13 countries), Asia (*n* = 24 from 8 countries), Europe (*n* = 21 from 16 countries), Latin America (*n* = 9 from 2 countries), North America (*n* = 9 from 3 countries), Oceania (*n* = 6 from 3 countries), and Central America (*n* = 1 from 1 county) (Table 1 and Table S3). These published reports of AHR were mostly (*n* > 6) from Brazil, India, and South Africa (Figure 2). During the study period (1996–2026), a total of 4840 small ruminant farms were assessed for resistance development in GINs to different single and co-formulations of anthelmintic compounds, of which 57% (2743) exhibited resistance.

Anthelmintic resistance was frequently reported in GINs infesting sheep (63.16%) than goats (36.84%). These animals were predominantly infected with resistant populations of *Haemonchus* spp. (43.28%) and *Trichostrongylus* spp. (24.56%) that were reported across 37 and 40 of the 46 countries encountered, respectively (Table 1). Anthelmintic compounds from seven drug classes have been reported to confer resistance in GINs. These GIN populations were most frequently reported to be resistant to benzimidazoles (35.01%), macrocyclic lactones (28.86%), and imidazothiazoles (19.43%) compounds. Resistance to these anthelmintic classes was reported in 41, 38, and 27 countries, respectively (Table 1 and Table 2). A co-formulation of benzimidazoles and imidazothiazoles (albendazole + levamisole) was also frequently associated with resistance in GINs, relative to other anthelmintic drug combinations (Table 1). The FECRT was the most frequently used method (80%), while the egg hatch test (11.48%), larval development test (8.19%), and molecular techniques (allele-specific polymerase chain reaction and deep amplicon sequencing polymerase chain reaction) (4.98%) were used less frequently (Table 1 and Table 2).

### 3.2. Pooled Prevalence Estimates, Subgroup Analyses, and Meta-Regression of Factors Associated with Anthelmintic Resistance in GINs of Small Ruminants

A total of 4809 GIN populations of small ruminant farms in 44 countries worldwide were included in the meta-analysis. The random-effects meta-analysis estimated an overall pooled prevalence for reported AHR of 64.60% (95% CI: 61.68–67.50), with considerable between-study heterogeneity (I^2^ = 77.00%, *p* < 0.0001) (Table 2). Visual inspection of the funnel plot (logit-transformed event rate versus standard error) showed no substantial asymmetry around the pooled effect estimate, suggesting a low risk of publication bias (Figure S1).

Subgroup meta-analysis was conducted to characterise AHR according to anthelmintic drug classes, GIN genera, host species, mode of administration, detection methods, and geography (Table 2 and Table 3). Gastrointestinal nematode resistance was frequently reported for benzimidazoles and macrocyclic lactones, with notably higher pooled prevalence estimates of 69.72% (95% CI: 65.23–73.86) and 68.60% (95% CI: 63.50–73.30), respectively (Table 2). Pooled prevalence estimates of reported resistance in GINs were 66.43% (95% CI: 49.89–79.73) for salicylanilides and 56.40% (95% CI: 49.80–65.18) for imidazothiazoles, with substantial between-study heterogeneity observed across studies (*p* < 0.0001; I^2^ = 64–75%). Relative to benzimidazoles, imidazothiazoles showed significantly lower odds of resistance (OR = 0.66; 95% CI: 0.47–0.92; *p* = 0.0143). In contrast, the odds of resistance development did not differ significantly between benzimidazoles and either macrocyclic lactones or salicylanilides (*p* > 0.05) (Table 3).

Among GIN genera, although *Haemonchus* spp. was the most frequently reported as resistant worldwide, *Oesophagostomum* spp. showed a relatively higher pooled prevalence estimate of reported AHR (71.70%; 95% CI: 59.10–81.60) (Table 2). The pooled prevalence of reported AHR was 69.80% (95% CI: 65.20–74.10), 64.90% (95% CI: 58.30–71.00), and 58.30% (95% CI: 50.00–66.10) for *Haemonchus* spp., *Trichostrongylus* spp., and *Teladorsagia* spp., respectively (Table 2). In the meta-regression analysis, it was noted that GIN genera were not a significant predictor of AHR prevalence. The odds of AHR in *Trichostrongylus* spp., *Teladorsagia* spp., and *Oesophagostomum* spp. did not differ statistically (*p* > 0.05) relative to *Haemonchus* spp. (Table 3).

The pooled prevalence estimates of reported AHR in GINs infecting sheep (64.79%; 95% CI: 61.20–68.22) and goats (64.10%; 95% CI: 58.37–69.19) were almost similar (Table 2). Additionally, the odds of resistance development (OR = 0.78) did not differ significantly (*p* > 0.05) between these small ruminants (Table 3). In the treatment of GINs in small ruminants, anthelmintic drugs were most frequently administered orally (64/94, 68%) (Table 2). However, a higher pooled prevalence of reported resistance was observed for subcutaneous administration (82.39%; 95% CI: 76.74–86.91) (Table 2). Nevertheless, the odds of resistance development in GINs did not differ significantly (*p* > 0.05) between subcutaneous and oral routes of drug administration (Table 3). In assessing AHR in GINs, the subgroup analysis revealed substantial differences among diagnostic approaches. Molecular techniques yielded a higher pooled prevalence estimate (93.55%; 95% CI: 79.34–98.21) and showed a considerable between-study homogeneity (I^2^ = 0; *p* = 0.91), although they were less frequently used for resistance detection. Studies that used FECRT, EHT, and LDT for resistance assessment reported pooled prevalence estimates of 63.95% (95% CI: 60.70–67.07), 64.89% (95% CI: 51.58–76.25), and 65.91% (95% CI: 53.69–76.33), respectively. Relative to the FECRT, the odds of resistance did not differ significantly from those observed for the EHT and LDT (*p* > 0.05) (Table 3). However, molecular detection methods were associated with significantly higher odds of resistance (OR = 4.04; 95% CI: 1.14–14.35; *p* = 0.0309).

Although Africa, Asia, and Europe accounted for more than 75% (71/94) of global reports of AHR in GINs of small ruminants, high pooled prevalence estimates were observed in Asia (73.01%; 95% CI: 67.47–77.93; I2 = 64.36, *p* < 0.0001) and Europe (63.62%; 95% CI: 57.39–69.42; I2 = 82.16, *p* < 0.0001), respectively. In contrast, studies from Africa showed a relatively lower pooled prevalence of 55.10% (95% CI: 48.19–61.83), with moderate between-study heterogeneity (I2 = 47.76%, *p* < 0.0001). Geographic region significantly influenced resistance prevalence. Compared with Africa (reference category), Oceania was associated with significantly lower odds of resistance (OR = 0.27; 95% CI: 0.18–0.43; *p* < 0.0001), whereas North America showed significantly higher odds (OR = 1.93; 95% CI: 1.12–3.33; *p* < 0.05) (Table 3). However, differences in odds of resistance for Asia (OR = 1.47), Europe (OR = 0.85), and Latin America (OR = 1.54), were not statistically significant (*p* > 0.05) (Table 3).

**Figure 3:**
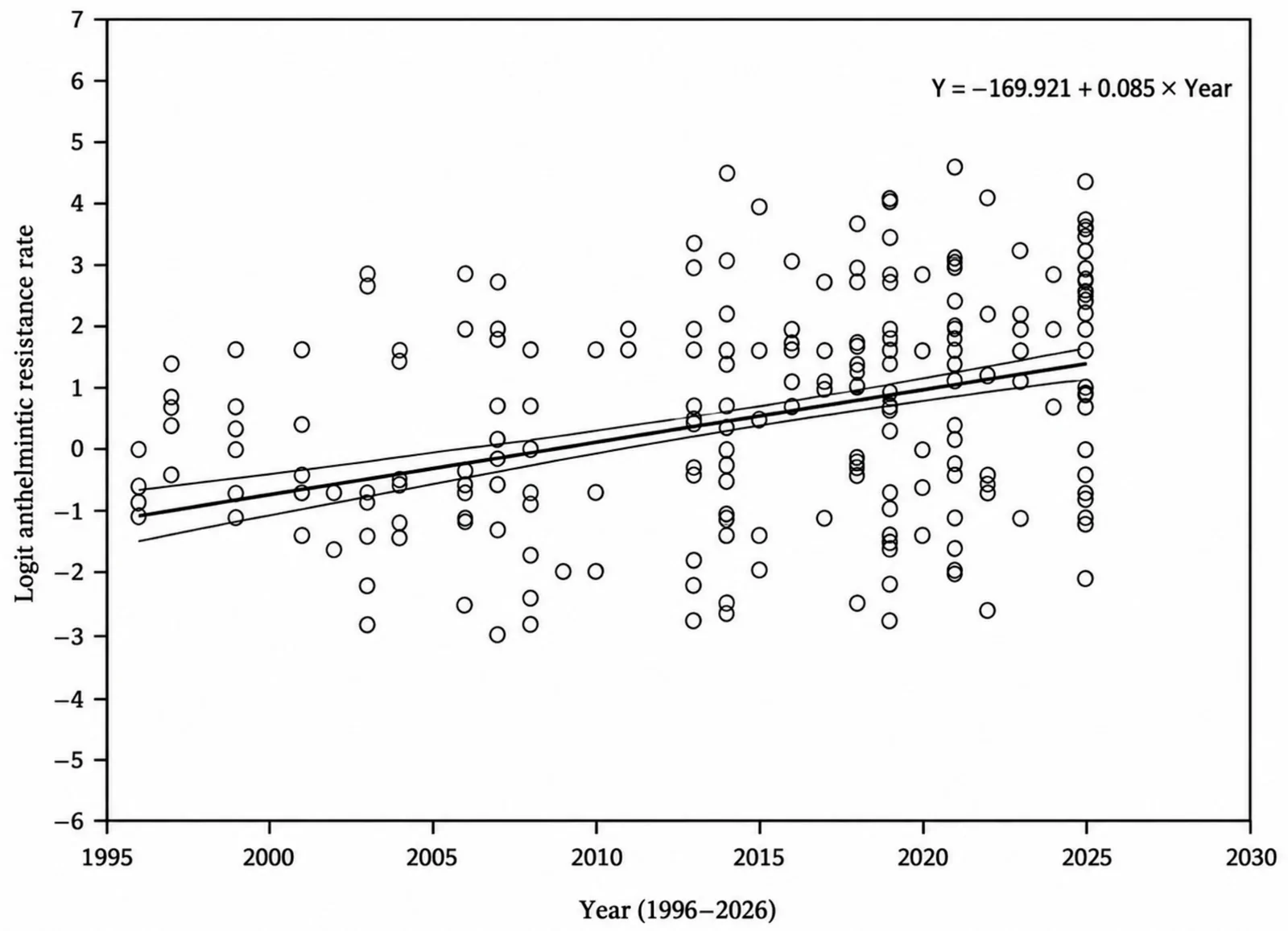
Rate of increase in the reported anthelmintic resistance prevalence in gastrointestinal nematode populations during the study period (1996–2026).

A meta-regression plot illustrating the association between year of study and reported AHR prevalence is shown in Figure 3, with circles representing individual study estimates weighted by inverse variance, while the shaded bands indicate the 95% confidence intervals. The meta-regression indicated a significant association between study year and reported AHR, with the odds of resistance increasing by approximately 9% per study year (OR = 1.09, *p* < 0.0001). The multivariable meta-regression model, which included study year, anthelmintic drug class, geographic region, and diagnostic method as moderators, accounted for 28% of the between-study variance in reported AHR prevalence (R^2^ = 0.28). However, substantial residual heterogeneity remained (τ^2^ = 1.44; I^2^ = 76.93%), indicating that a large proportion of the variability was not explained by the moderators included in the model.

## 4. Discussion

This study provides a comprehensive synthesis of the global prevalence of AHR in GINs infecting small ruminants and highlights the influence of study-level factors on the prevalence of resistance. Anthelmintic resistance has been reported in GIN populations infecting ruminants worldwide against all major classes of anthelmintic drugs [32,41]. In large ruminants, Mederos et al. [48] reported an overall pooled prevalence of 72.0% for AHR in cattle GINs. Similarly, the present study found that approximately two-thirds (64.60%) of GIN populations infecting small ruminants worldwide exhibited AHR, although the prevalence varied considerably among the included studies. These findings indicate that AHR in GINs of small ruminants is no longer an emerging problem but a well-established global challenge. Indiscriminate anthelmintic drug use over several decades may have contributed to this problem by exerting selection pressure on GIN populations and increasing the frequency of resistance alleles [49].

A pattern consistent with the findings of Saidu et al. [5] was also observed in the present study, with the majority of published reports on AHR in GIN of small ruminants originating from countries in Europe, Africa, and Asia. This geographical distribution reflects the availability of published evidence and should not be interpreted as indicating a higher prevalence of AHR in these regions. The high number of published reports from Europe may reflect the region’s well-established veterinary research institutions and long-term surveillance programmes [50,51]. These developments have improved access to advanced diagnostic methods, including molecular techniques, facilitating the detection and reporting of AHR [50]. In addition, the widespread involvement of veterinarians in designing parasite control programmes and the routine use of coprological analyses by small-ruminant farmers may have further enhanced the identification and monitoring of resistant GIN populations [52]. In several developing countries across Africa and Asia, research on AHR in small ruminants has increased because GINs are major constraints to livestock production [32,53]. Brazil, India, and South Africa are among the countries with the largest sheep and goat populations globally. Small ruminant production systems in these countries play a vital role in food security, rural livelihoods, and national economies [35,54,55], underscoring the importance of effective parasite control strategies [10]. Most small ruminant farms in these countries operate under communal grazing systems, where farmers often have unrestricted access to anthelmintic drugs without a veterinary prescription. The repeated use of the same anthelmintic class with little or no rotation, coupled with the interaction of treated and untreated animals, may facilitate the transmission and spread of anthelmintic-resistant GINs [2,32,56,57]. Furthermore, the warm and humid climatic conditions in many African and Asian countries may favour the survival and year-round transmission of GIN larvae [58], potentially resulting in more frequent deworming and greater selection pressure for AHR.

The lower number of published reports from the Americas and Oceania may reflect differences in evidence availability and database coverage rather than a genuinely lower prevalence of AHR [59]. Studies from these regions may be underrepresented in international databases because some findings are published in local or non-indexed journals, while language restrictions may have further limited the retrieval of studies from Latin America. The lower odds of reported AHR in Oceania observed in the present study may partly reflect differences in parasite control practices, including the use of faecal egg count monitoring, targeted selective treatment, and strategies aimed at preserving anthelmintic efficacy [60,61]. Similarly, the higher odds observed in North America may reflect differences in the historical and contemporary use of anthelmintic classes, treatment practices, production systems, and parasite-management strategies [62,63,64]. However, these regional associations should be interpreted cautiously because they may also reflect differences in diagnostic methods, study periods, GIN genera, anthelmintic classes, sampling strategies, and research intensity across regions. Thus, geographic region should be regarded as an indicator of broader study and production-level differences rather than as an independent determinant of AHR prevalence.

Host species (sheep or goats) was not a significant predictor of AHR in the meta-regression. However, the present study also found that assessments of AHR in GINs were conducted more frequently in sheep than in goats, consistent with the findings of Macedo et al. [65]. This pattern may reflect differences in research priorities and the economic importance of sheep production in many countries, which may have contributed to greater research and surveillance efforts in sheep than in goats [66,67]. In addition, the greater susceptibility of sheep to GIN infections may have contributed to their greater representation in parasite control and AHR monitoring programmes [68].

The lower number of AHR assessments in goats may also reflect differences in anthelmintic availability and registration, as fewer anthelmintic compounds are registered for use in goats in some countries [69]. Although goats are often regarded as being at greater risk of developing AHR because of pharmacokinetic differences and management practices, these host-specific effects are reduced when sheep and goats are managed together under the same production system and parasite control strategies [70]. Both host species are infected by similar genera of GINs, which are frequently transmitted through shared grazing pastures, facilitating the exchange of resistant parasite populations between hosts [70].

The frequent representation of *Haemonchus* spp., *Oesophagostomum* spp., and *Trichostrongylus* spp. among reports of AHR is consistent with the findings of Allaie et al. [71], who similarly identified these genera as commonly associated with AHR in small-ruminant production systems. Their frequent occurrence may partly reflect shared biological and epidemiological characteristics that facilitate persistence and transmission. These nematodes have direct life cycles, eliminating the need for intermediate hosts and enabling transmission through ingestion of infective third-stage larvae on contaminated pasture [71,72]. Their infective larvae can also persist under favourable environmental conditions, increasing opportunities for host exposure during grazing [73,74]. In addition, *Haemonchus* spp. have exceptionally high fecundity, producing approximately 1000–10,000 eggs per day, and can undergo hypobiosis, which may enhance their persistence under unfavourable environmental conditions [75].

The absence of a significant genus effect in the present meta-regression suggests that differences in reported AHR may not be attributable to GIN genus alone. Because multiple GIN genera commonly occur within the same host populations and may be exposed to similar anthelmintic treatment and management practices, comparable selection pressures could contribute to overlapping resistance patterns across genera. However, this interpretation remains hypothetical, as the present study did not directly assess within-host co-infections, shared exposure histories, or the development of resistance within individual parasite populations. Other factors, including anthelmintic class, treatment practices, diagnostic methods, geographic location, and study characteristics, may also contribute to the observed variation in resistance estimates [36,76].

Resistance to benzimidazoles, macrocyclic lactones, and salicylanilides is widespread among GINs infecting small ruminants [32,37]. In this study, these anthelmintic drug classes were noted to exhibit high odds of resistance. The long history of use of these anthelmintic drug classes has provided substantial time for the selection and dissemination of resistance-associated alleles within GIN populations of small ruminants. However, at the global level, differences in the availability, registration, and use of anthelmintic drugs among countries may have resulted in unequal exposure to specific drug classes. Benzimidazoles were introduced during the 1960s, and resistance to benzimidazoles was reported shortly thereafter in sheep [77] and goats [78] across several GIN species [76]. Although different benzimidazole products are available, they share the same mode of action. Consequently, livestock farmers who rotate or substitute anthelmintics within the benzimidazole class, rather than between different chemical classes, may continue to exert similar selection pressure on GINs, thereby promoting the persistence and spread of benzimidazole-resistant populations [32,79]. Macrocyclic lactones were introduced in the early 1980s [70], and resistance subsequently emerged in 1987 among several economically important GINs of small ruminants [35]. The use of macrocyclic lactones for the control of ectoparasites may broaden selection pressure across parasite populations due to the systemic activity of this drug class [32,80]. Salicylanilides are narrow-spectrum anthelmintics that have been widely used in small-ruminant production systems since their introduction in the 1970s, with resistance subsequently reported in several countries [35,81,82]. Previous studies have suggested that increased reliance on closantel, particularly in settings where resistance to other anthelmintic classes is widespread, may increase selection pressure for salicylanilide-resistant GIN populations [30,82,83]. Closantel is a salicylanilide compound with activity primarily against blood-feeding GINs, particularly *Haemonchus contortus*, and is commonly formulated or administered in combination with benzimidazole or macrocyclic lactone compounds [30,82]. Consequently, where resistance to these other drug classes is prevalent, greater reliance on closantel for *H. contortus* control could potentially increase selection pressure for salicylanilide resistance. However, closantel exposure was not assessed as a moderator in the present meta-analysis. Therefore, its contribution to the observed resistance prevalence cannot be established. This mechanism remains a plausible explanation based on previous evidence and warrants further investigation.

The lower odds of resistance to imidazothiazoles observed in the present study are consistent with the findings of Baudinette et al. [37], who similarly reported relatively low levels of resistance in GINs infecting goats. Unlike benzimidazoles, macrocyclic lactones, and salicylanilides, resistance to imidazothiazoles primarily involves alterations in the nicotinic acetylcholine receptors, a mechanism that appears to be genetically more complex and has been reported less frequently than resistance mechanisms associated with other anthelmintic classes [84]. Although levamisole, the only commercially available imidazothiazole, has been widely used in small ruminant production systems for approximately six decades [85], resistance to this drug has generally remained less prevalent than resistance to benzimidazoles and macrocyclic lactones [37]. Several mechanisms reported in the literature may potentially explain the comparatively lower odds of resistance observed in the present study. First, resistance is generally inherited as a partially recessive trait, requiring nematodes to inherit resistance alleles from both parents to survive treatment, whereas heterozygous individuals remain susceptible. As a result, resistance alleles accumulate more slowly within parasite populations than would be expected under dominant inheritance [86]. Second, levamisole is rapidly eliminated from the host, resulting in a relatively short period of drug exposure and reduced selection pressure for resistant genotypes compared with more persistent anthelmintics [34]. Finally, resistance-associated mutations may impose fitness costs, such as reduced survival or reproductive performance in the absence of drug treatment, thereby limiting the persistence and spread of resistance alleles within parasite populations [85].

Although anthelmintics are predominantly administered orally, a higher frequency of AHR was observed among animals treated by the subcutaneous route, despite the mode of administration not being a significant predictor of resistance. Pharmacokinetic differences between administration routes may partly explain this observation. Subcutaneous formulations generally exhibit slower absorption and elimination than orally administered drugs, resulting in prolonged persistence of the drug within the host. As drug concentrations decline, GINs may be exposed to extended periods of subtherapeutic concentrations, allowing partially resistant individuals to survive treatment and increasing selection pressure for resistant parasite populations [79,87,88]. However, the observed difference may also be influenced by other factors, including drug class, formulation, country, and differences in study design.

Our findings indicate that diagnostic methodology contributes to variation in reported resistance prevalence and provides an important qualification for the interpretation of the overall pooled estimate. The higher pooled prevalence estimates and odds of reported AHR associated with molecular techniques are likely attributable to their greater sensitivity than conventional phenotypic assays [29,89]. Molecular techniques provide a more direct measure of resistance at the worm level because AHR is genetically determined. However, only a limited number of validated genetic markers for AHR are currently available. Unlike phenotypic methods, molecular techniques identify specific genetic mutations associated with resistance and can detect resistance alleles even when they occur at low frequencies within GIN populations [90]. However, the detection of resistance-associated alleles is not necessarily equivalent to phenotypically expressed resistance. Consequently, they enable the detection of emerging resistance before it becomes phenotypically apparent or compromises treatment efficacy [31,91]. In contrast, conventional in vivo methods, such as the FECRT, assess resistance through reductions in anthelmintic efficacy and may fail to detect resistance at an early stage before measurable treatment failure becomes apparent [92].

The meta-regression analysis showed that study year was significantly associated with AHR, with the odds of reported resistance increasing by 9% per year. An increase in AHR over time has also been reported by Bull et al. [93] among GINs on sheep farms between 2013 and 2016, although their findings were based on a specific population and study period. The progressive increase in resistance over time is likely driven by sustained selection pressure resulting from repeated anthelmintic use, which favours the survival and reproduction of resistant parasites while eliminating susceptible individuals. Consequently, resistance alleles accumulate and become increasingly prevalent within GIN populations over successive generations [94]. This evolutionary process is further accelerated by the short generation time (approximately 21–30 days) and high fecundity of many GIN species, allowing resistant genotypes to spread rapidly through parasite populations [95]. However, the observed temporal increase in AHR should be interpreted with caution, because the detection of resistance depends on when and how frequently it is assessed and reported.

The meta-regression model explained 28% of the between-study variance in reported AHR prevalence, indicating that study year, anthelmintic drug class, geographic region, and diagnostic method accounted for a measurable but limited proportion of the observed heterogeneity [80,96]. Substantial residual heterogeneity remained (τ^2^ = 1.44; I^2^ = 76.93%), indicating that most between-study variability was not explained by the moderators included in the model. This residual heterogeneity may reflect differences in farm management, anthelmintic treatment history and dosing practices, grazing systems, refugia management, parasite population structure, environmental conditions, and other methodological characteristics [59,97]. Several potentially relevant determinants were also inconsistently reported across studies and therefore could not be incorporated into the meta-regression. Thus, the identified moderators should be interpreted as factors associated with variation in reported AHR prevalence rather than as comprehensive explanations of the observed heterogeneity.

A further limitation is that some studies contributed multiple class-specific resistance estimates, which may have been derived from overlapping farms or GIN populations. Because sufficient information to quantify the dependence among these estimates was not consistently available, within-study correlation could not be formally modelled. Consequently, some effect sizes may not have been statistically independent, which should be considered when interpreting the pooled and subgroup estimates. Future studies should standardise the reporting of treatment practices, refugia management, grazing systems, environmental conditions, and parasite genetic characteristics to facilitate more comprehensive risk-factor analyses and improve the comparability of global AHR estimates. A formal risk of bias assessment was not performed because of the methodological heterogeneity of the included studies and the lack of a single assessment tool applicable across all study designs.

## 5. Concluding Remarks

Overall, the findings indicate that reported anthelmintic resistance in gastrointestinal nematodes of small ruminants is widespread among the populations represented in the available literature. The pooled prevalence was 64.60%, with substantial between-study heterogeneity, and resistance prevalence varied according to anthelmintic class, diagnostic method, geographic region, and study year. The meta-regression identified a 9% annual increase in the odds of reported resistance; however, this represents a study-level temporal association and should not be interpreted as evidence of causation. The substantial residual heterogeneity further indicates that important sources of variation in resistance remain unexplained. In light of these findings and evidence from the broader literature, sustainable AHR management should incorporate integrated approaches, including targeted selective treatment, refuge-based strategies, molecular surveillance, host genetic selection, biological control, nutritional management, and validated plant-based interventions. These approaches were not evaluated in the present meta-analysis but represent complementary strategies that warrant consideration within evidence-based anthelmintic stewardship and integrated parasite management programmes.

## Figures and Tables

**Table 1:** Characteristics of studies included in the systematic review.

Factor	Sub-Category	Studies Reporting Resistance (%)	No of Countries	List of Countries
Anthelmintic drug class (Total No of studies = 257)	BZ	90 (35.01)	41	Kenya, Zimbabwe, South Africa, Tanzania, Mozambique, Algeria, Ethiopia, Egypt, Uganda, Nigeria, Pakistan, Iraq, India, Malaysia, Bangladesh, Thailand, Jordan, New Zealand, Australia, Fiji, Spain, Denmark, France, Greece, Lithuania, Netherlands, Poland, Belgium, Romania, Czech Republic, Latvia, Sweden, Germany, Italy, Brazil, Colombia, Georgia, Canada, Mexico, Trinidad, Costa Rica
	ML	74 (28.79)	38	Zimbabwe, South Africa, Tanzania, Algeria, Ethiopia, Uganda, Botswana, Turkey, Cameroon, Nigeria, Pakistan, Iraq, India, Malaysia, Thailand, Jordan, New Zealand, Australia, Fiji, Italy, Spain, Denmark, France, Lithuania, Netherlands, Poland, Belgium, Romania, Czech Republic, Latvia, Sweden, Germany, Brazil, Colombia, Georgia, Canada, Mexico, Costa Rica
	IMD	55 (21.40)	27	Kenya, Zimbabwe, South Africa, Tanzania, Mozambique, Ethiopia, Uganda, Nigeria, Pakistan, Iraq, India, Malaysia, Thailand, Jordan, New Zealand, Australia, Fiji, Italy, Greece, Poland, Brazil, Colombia, Georgia, Canada, Mexico, Trinidad, Costa Rica
	SLD	13 (5.06)	5	Brazil, Mexico, Ethiopia, South Africa, Zimbabwe
	OP	3 (1.17)	3	South Africa, Australia, Brazil
	AAD	3 (1.17)	2	Brazil, Netherlands
	THP	1 (0.39)	1	India
	BZ + IMD	5 (1.94)	4	New Zealand, Brazil, Australia, Fiji
	IMD + SLD	2 (0.99)	2	Tanzania, Italy
	BZ + SLD	2 (0.99)	2	Italy, South Africa
	BZ + ML	2 (0.99)	2	Thailand, Latvia
	IMD + ISN	1 (0.39)	1	South Africa
	IMD + ML	1 (0.39)	1	Pakistan
	BZ + IMD + ML + SLD	1 (0.39)	1	Australia
	SLD + ML	1 (0.39)	1	Australia
	ML + BZ + IMD	1 (0.39)	1	Australia
	OP + BZ + IMD	1 (0.39)	1	Australia
	OP + ML	1 (0.39)	1	Australia
Bioassay technique (Total No of studies = 122)	FECRT	98 (80.33)	46	Kenya, Zimbabwe, South Africa, Zambia, Mozambique, Tanzania, Algeria, Ethiopia, Uganda, Botswana, Egypt, Nigeria, Cameroon, Pakistan, Turkey, Iraq, India, Malaysia, Bangladesh, Thailand, Jordan, New Zealand, Australia, Fiji, Georgia, Italy, Spain, Denmark, Switzerland, France, Greece, Lithuania, Netherlands, Poland, Belgium, Romania, Czech Republic, Latvia, Sweden, Germany, Brazil, Colombia, Trinidad, Canada, Mexico, Costa Rica.
	EHT	14 (11.48)	11	South Africa, Pakistan, India, Thailand, Spain, Denmark, Lithuania, Poland, Romania, Czech Republic, Mexico
	LDT	10 (8.19)	11	South Africa, India, Iraq, Australia, Brazil, Lithuania, Poland, Romania, Canada, Spain, Mexico
	PCR	6 (4.92)	7	South Africa, Egypt, Thailand, India, Mexico, Brazil, Spain
	MALDT	2 (1.64)	2	Canada, South Africa
Host species (Total No of studies = 114)	Sheep	72 (63.16)	41	Kenya, Zimbabwe, South Africa, Zambia, Mozambique, Tanzania, Algeria, Ethiopia, Uganda, Egypt, Nigeria, Cameroon, Pakistan, Turkey, Iraq, India, Bangladesh, Thailand, Jordan, New Zealand, Australia, Georgia, Italy, Spain, Denmark, Switzerland, France, Greece, Lithuania, Netherlands, Poland, Belgium, Romania, Czech Republic, Latvia, Sweden, Germany, Brazil, Canada, Mexico, Costa Rica
	Goat	42 (36.84)	36	Kenya, Zimbabwe, South Africa, Mozambique, Tanzania, Ethiopia, Uganda, Botswana, Egypt, Nigeria, Cameroon, Pakistan, Iraq, India, Malaysia, Thailand, Jordan, Australia, Fiji, Georgia, Italy, Denmark, Switzerland, France, Lithuania, Poland, Romania, Czech Republic, Latvia, Germany, Brazil, Colombia, Canada, Mexico, Costa Rica
GIN genera (Total No of studies = 171)	*Haemonchus* sp.	74 (43.28)	37	Kenya, Zimbabwe, South Africa, Zambia, Mozambique, Tanzania, Ethiopia, Uganda, Botswana, Egypt, Nigeria, Cameroon, Pakistan, Turkey, Iraq, India, Bangladesh, Thailand, Jordan, New Zealand, Australia, Italy, Georgia, Denmark, Switzerland, Netherlands, Belgium, Latvia, Sweden, Germany, Poland, Brazil, Colombia, Trinidad, Canada, Mexico, Costa Rica
	*Trichostrongylus* spp.	42 (24.56)	40	Kenya, Zimbabwe, South Africa, Mozambique, Tanzania, Algeria, Uganda, Botswana, Nigeria, Cameroon, Pakistan, Turkey, Iraq, India, Bangladesh, Thailand, Jordan, New Zealand, Australia, Italy, France, Greece, Lithuania, Netherlands, Belgium, Poland, Brazil, Colombia, Mexico, Costa Rica, Canada, Sweden, Germany, Spain, Switzerland, Denmark, Latvia, Romania, Georgia, Malaysia, Fiji
	*Teladorsagia* spp.	24 (14.04)	22	South Africa, Tanzania, Algeria, Italy, Spain, New Zealand, Australia, France, Greece, Lithuania, Netherlands, Belgium, Poland, Sweden, Denmark, Latvia, Romania, Georgia, Switzerland, Germany, Iraq, Turkey
	*Oesophagostomum* spp.	11 (6.43)	10	Mozambique, Tanzania, Uganda, Nigeria, Pakistan, Italy, Greece, Costa Rica, Mexico, Brazil
	*Cooperia* spp.	5 (2.92)	6	Pakistan, Canada, Mexico, Costa Rica, Colombia, Brazil
	*Nematodirus* spp.	4 (2.34)	3	Iraq, New Zealand, Spain
	*Chabertia* spp.	3 (1.75)	3	India, Costa Rica, Spain
	*Strongyloides* spp.	3 (1.75)	1	India
	*Trichuris* spp.	2 (1.17)	2	Ethiopia, Thailand
	*Ostertagia* spp.	2 (1.17)	2	Australia, Canada
	*Marshalligia* sp.	1 (0.58)	1	Iraq
Geographic location (Total No of studies = 105)	Africa	35 (33.33)	13	Algeria, Botswana, Cameroon, Egypt, Ethiopia, Kenya, Mozambique, Nigeria, South Africa, Tanzania, Uganda, Zambia, Zimbabwe
	Asia	24 (22.86)	8	Bangladesh, India, Iraq, Jordan, Malaysia, Pakistan, Thailand, Turkey
	Europe	21 (20)	16	Belgium, Czech Republic, Denmark, France, Georgia, Germany, Greece, Italy, Latvia, Lithuania, Netherlands, Poland, Romania, Spain, Sweden, Switzerland
	Latin America	9 (8.57)	2	Brazil, Colombia
	North America	9 (8.57)	3	Canada, Mexico, Trinidad
	Oceania	6 (5.71)	3	Australia, Fiji, New Zealand
	Central America	1 (0.95)	1	Costa Rica
	Total	105	46	

Abbreviations: AAD, amino-acetonitrile derivative; OP, organophosphate; NTS, nitrophenol substitutes; THP, Tetrahydropyrimidine; SLDs, Salicylanilides; BZ + IMD, coformulation of Benzimidazoles + Imidazothiazoles; IMD + SLD, coformulation of Imidazothiazoles + Salicylanilides; BZ + SLD, coformulation of Benzimidazoles + Salicylanilides; BZ + ML, coformulation of Benzimidazoles + Macrocyclic lactones; IMD + ISN, coformulation of Imidazothiazoles + Isoquinoline derivatives; IMD + ML, coformulation of Imidazothiazoles + Macrocyclic lactones; BZ + IMD + ML + SLD, coformulation of Benzimidazoles + Imidazothiazoles + Macrocyclic lactones + Salicylanilides; SLD + ML, coformulation of Salicylanilides + Macrocyclic lactones; ML + BZ + IMD, coformulation of Macrocyclic lactones + Benzimidazoles + Imidazothiazoles; OP + BZ + IMD, coformulation of Organophosphates + Benzimidazoles + Imidazothiazoles; OP + ML, coformulation of Organophosphates + Macrocyclic lactones; GIN, gastrointestinal nematodes; FECRT, faecal egg count reduction test; LDT, larval development test; EHT, egg hatch test; TBZ, thiabendazole; DD, discrimination dose; MALDT, micro-agar larval development test.

**Table 2:** Overall pooled prevalence estimates (PPE) and subgroup analysis of AHR in gastrointestinal nematode populations of small ruminants globally during the study period (1996–2026).

Factor	Variable	No of Countries	Studies Included	No of Trials	No of Farms Assessed	No of Farms with AHR	PPE (%) (95%CI)	Measures of Heterogeneity		
								Q-Value	I^2^ (%)	*p*-Value
Overall PPE	-	44	94	557 *	4809	2712	64.60 (61.60–67.50)	2423.615	77.00	<0.0001
Anthelmintic drugs classes ^b^	BZ	39	83	222	1827	1153	69.72 (65.23–73.86)	857.427	75.11	<0.0001
	ML	39	68	168	17,405	1059	68.60 (63.50–73.30)	831.559	79.68	<0.0001
	IMD	26	47	104	711	376	56.40 (49.80–65.18)	303.384	66.05	<0.0001
	SLD ^a^	4	13	19	145	101	66.43 (49.89–79.73)	50.604	64.43	<0.0001
	AAD ^a^	2	3	10	53	12	29.28 (15.25–48.79)	-	-	-
	OP ^a^	2	2	3	15	2	37.95 (07.78–81.61)	-	-	-
	THP ^a^	1	1	1	25	3	12.00 (10.11–64.53)	-	-	-
	BZ + IMD	4	5	10	191	48	18.23 (9.02–33.39)	129.785	93.065	<0.0001
	BZ + SLD ^a^	3	4	5	67	42	88.80 (32.30–99.22)	-	-	-
	IMD + SLD ^a^	2	2	3	41	27	96.19 (75.51–99.52)	-	-	-
	BZ + ML ^a^	2	2	3	15	12	86.72 (46.14–98.03)	-	-	-
	IMD + ML ^a^	1	1	1	15	15	96.88 (45.07–99.91)	-	-	-
	OP + ML ^a^	1	1	2	17	1	5.90 (7.15–35.17)	-	-	-
	OP + BZ + IMD ^a^	1	1	2	33	6	18.18 (3.76–55.82)	-	-	-
	ML + BZ + IMD ^a^	1	1	2	6	1	16.67 (2.12–64.83)	-	-	-
	BZ + IMD + ML + SLD ^a^	1	1	1	20	4	2.00 (1.93–76.04)	-	-	-
	SLD + ML ^a^	1	1	1	18	6	37.50 (04.67–88.03)	-	-	-
GIN genera ^b^	*Haemonchus* sp.	36	66	224	2660	1703	69.80 (65.20–74.1)	767.543	70.95	<0.0001
	*Trichostrongylus* spp.	26	44	133	2083	1085	64.90 (58.30–71.00)	660.195	80.01	<0.0001
	*Teladorsagia* spp.	20	23	69	1081	642	58.30 (5.00–66.10)	329.555	79.37	<0.0001
	*Oesophagostomum* spp.	12	8	34	365	253	71.70 (59.10–81.60)	103.468	68.11	<0.0001
	*Cooperia* spp.	4	5	12	470	207	53.20 (34.70–70.90)	82.258	86.62	<0.0001
	Not mentioned	9	12	50	565	286	55.60 (45.20–65.50)	153.871	68.16	<0.0001
	*Nematodirus* spp. ^a^	3	4	10	535	171	39.70 (25.70–55.70)	-	-	-
	*Chabertia* spp. ^a^	3	3	8	134	68	71.90 (29.70–93.90)	-	-	-
	*Strongyloides* spp. ^a^	2	3	8	21	20	73.00 (52.50–86.90)	-	-	-
	*Ostertagia* spp. ^a^	1	1	5	320	88	28.60 (16.10–45.70)	-	-	-
	*Trichuris* spp. ^a^	2	2	3	20	16	87.50 (55.90–97.50)	-	-	-
	*Marshalligia* sp. ^a^	1	1	1	6	4	66.70 (26.80–91.60)	-	-	-
Host species ^b^	Sheep	33	66	393	3082	1723	64.79 (61.20–68.22)	1810.47	78.24	<0.0001
	Goat	21	38	164	1118	782	64.10 (58.37–69.19)	578.739	71.835	<0.0001
Mode of administration	Oral	29	64	303	1597	1158	67.11 (63.19–70.82)	822.234	63.27	<0.0001
	Subcutaneous	17	28	81	534	398	82.39 (76.74–86.91)	333.329	76.00	<0.0001
	Not specified	16	17	101	1157	898	39.00 (33.33–44.99)	599.099	82.97	<0.0001
	Not applicable	14	17	65	735	464	67.87 (60.17–74.70)	297.961	78.52	<0.0001
	Pour- on ^a^	4	4	7	72	68	92.43 (80.49–97.31)	-	-	-
Detection Method ^b^	FECRT	43	91	490	3876	2087	63.95 (60.70–67.07)	2087.957	76.44	<0.0001
	EHT	10	14	26	248	147	64.89 (51.58–76.25)	100.121	75.03	<0.0001
	LDT	8	10	31	290	181	65.91 (53.69–76.33)	172.324	82.59	<0.0001
	PCR	5	6	7	247	175	93.55 (79.34–98.21)	2.074	0.00	0.91
	MALDT ^a^	2	2	3	26	15	56.21 (22.06–85.34)	-	-	-
Region	Africa	12	26	112	684	326	55.10 (48.19–61.83)	212.470	47.76	<0.0001
	Asia	7	23	131	751	540	73.01 (67.47–77.93)	367.594	64.36	<0.0001
	Europe	16	22	116	1208	688	63.62 (57.39–69.42)	650.365	82.16	<0.0001
	Latin America	2	8	75	508	346	76.86 (70.15–82.44)	220.567	65.99	<0.0001
	North America	3	8	51	487	375	79.91 (71.98–86.03)	176. 135	71.61	<0.0001
	Oceania	3	5	60	1029	336	30.02 (23.73–37.16)	295.526	80.04	<0.0001
	Central America ^a^	1	1	12	142	101	90.79 (81.74–95.60)	-	-	-

Abbreviations: AAD, amino-acetonitrile derivative; NTS, nitrophenol substitutes; THP, tetrahydropyrimidine; OP, organophosphate; SLD, salicylanilides; BZ + IMD, coformulation of benzimidazoles + imidazothiazoles; IMD + SLD, coformulation of imidazothiazoles + salicylanilides; BZ + SLD, coformulation of benzimidazoles + salicylanilides; BZ + ML, coformulation of benzimidazoles + macrocyclic lactones; IMD + ML, coformulation of imidazothiazoles + macrocyclic lactones; BZ + IMD + ML + SLD, coformulation of benzimidazoles + imidazothiazoles + macrocyclic lactones + salicylanilides; SLD + ML, coformulation of salicylanilides + macrocyclic lactones; ML + BZ + IMD, coformulation of macrocyclic lactones + benzimidazoles + imidazothiazoles; OP + BZ + IMD, coformulation of organophosphates + benzimidazoles + imidazothiazoles; OP + ML, coformulation of organophosphates + macrocyclic lactones; GIN, gastrointestinal nematodes; FECRT, faecal egg count reduction test; LDT, larval development test; EHT, egg hatch test; TBZ, thiabendazole; DD, discrimination dose; MALDT, micro-agar larval development test. * Each reported chemical class, GIN genera, host species and detection method was treated as a separate observation within the dataset. ^a^ Measures of heterogeneity for variables with fewer than 5 included studies were omitted. ^b^ Multiple studies reported more than one anthelmintic drug class, GIN genera, host species, mode of administration, and detection method.

**Table 3:** Meta-regression analysis of study-level factors associated with anthelmintic resistance in gastrointestinal nematode populations.

Factor	Comparison/Unit	Logit Coefficient (β)	Odds Ratio (OR)	95% CI (OR)	*p*-Value
Year of study	Per 1-year increase	0.085	1.09	1.07 to 1.10	<0.0001
Anthelmintic drug class	Imidazothiazoles	−0.3838	0.68	0.48 to 0.95	0.0241
	Macrocyclic lactones	0.1310	1.14	0.84 to 1.54	0.3918
	Salicylanilides	−0.0194	0.98	0.45 to 1.95	0.9559
GIN genera	*Trichostrongylus* spp.	−0.0957	0.91	0.67 to 1.21	0.5238
	*Teladorsagia* spp.	−0.0327	0.98	0.66 to 1.41	0.8649
	*Oesophagostomum* spp.	−0.0833	0.92	0.54 to 1.56	0.7563
Host species	Goat	−0.1481	0.86	0.633 to 1.17	0.3461
Mode of administration	Subcutaneous	0.1584	1.17	0.73 to 1.86	0.5062
Detection method	EHT	−0.1811	0.83	0.47 to 1.46	0.5264
	LDT	−0.4326	0.64	0.38 to 1.09	0.1049
	Molecular techniques	1.396	4.04	1.14 to14.35	0.0309
Region	Asia	0.3844	1.47	0.97 to 2.20	0.0635
	Europe	−0.1669	0.85	0.55 to 1.28	0.4288
	Latin America	0.4347	1.54	0.97 to 2.46	0.0673
	North America	0.6571	1.93	1.12 to 3.33	0.0182
	Oceania	−1.3135	0.27	0.17 to 0.41	<0.0001
	Model statistics		Value		
	R^2^ (between-study variance explained)		0.28		
	*τ*^2^ (residual between study variance)		1.44		
	I^2^ (residual heterogeneity%)		76.93		

Footnotes: 1. Odds ratios were derived by exponentiating the log regression coefficients (OR = e^β). Odds ratios represent comparisons with reference categories: Benzimidazole (chemical class), Feacal egg count reduction test (detection method), *Haemonchus* spp. (GIN genera), sheep (host species) and Africa (region). 2. OR > 1 indicates increased odds of anthelmintic resistance, and OR < 1 indicates reduced odds of detected resistance. 3. Negative β coefficients (logit scale) indicate lower odds of detected resistance, and positive β coefficients (logit scale) indicate higher odds of detected resistance. 4. R2 represents the proportion of between-study variance in reported anthelmintic resistance in the GIN populations explained by the meta-regression model. τ2 and I2 reflect the level of residual heterogeneity remaining after adjustment for the included moderators.

## Data Availability

No new data were created or analyzed in this study. Data sharing is not applicable to this article.

## Supplementary Materials

The following supporting information can be downloaded at https://www.mdpi.com/article/10.3390/vetsci13101077/s1, Figure S1. Funnel plot for assessment of publication bias; Table S1. PRISMA checklist; Table S2. Search strategies used to identify studies reporting AHR in GINs of small ruminants; Table S3. List of studies on anthelmintic resistance (AHR) in gastrointestinal nematodes of small ruminants globally during the study period (1996–2026) [98,99,100,101,102,103,104,105,106,107,108,109,110,111,112,113,114,115,116,117,118,119,120,121,122,123,124,125,126,127,128,129,130,131,132,133,134,135,136,137,138,139,140,141,142,143,144,145,146,147,148,149,150,151,152,153,154,155,156,157,158,159,160,161,162,163,164,165,166,167,168,169,170,171,172,173,174,175,176,177,178,179,180,181,182,183].
