# Supplementary material for: Patterns of Anthelmintic Resistance in Gastrointestinal Nematodes of Small Ruminants: A Systematic Review and Meta-Analysis
Source: Vet Sci. 2026 Oct 12;13(10):1077. doi: 10.3390/vetsci13101077 (PMC13709475; doi:10.3390/vetsci13101077)
Supplement: Supplementary file 1 [file vetsci-13-01077-s001.zip › Figure S1.pdf]

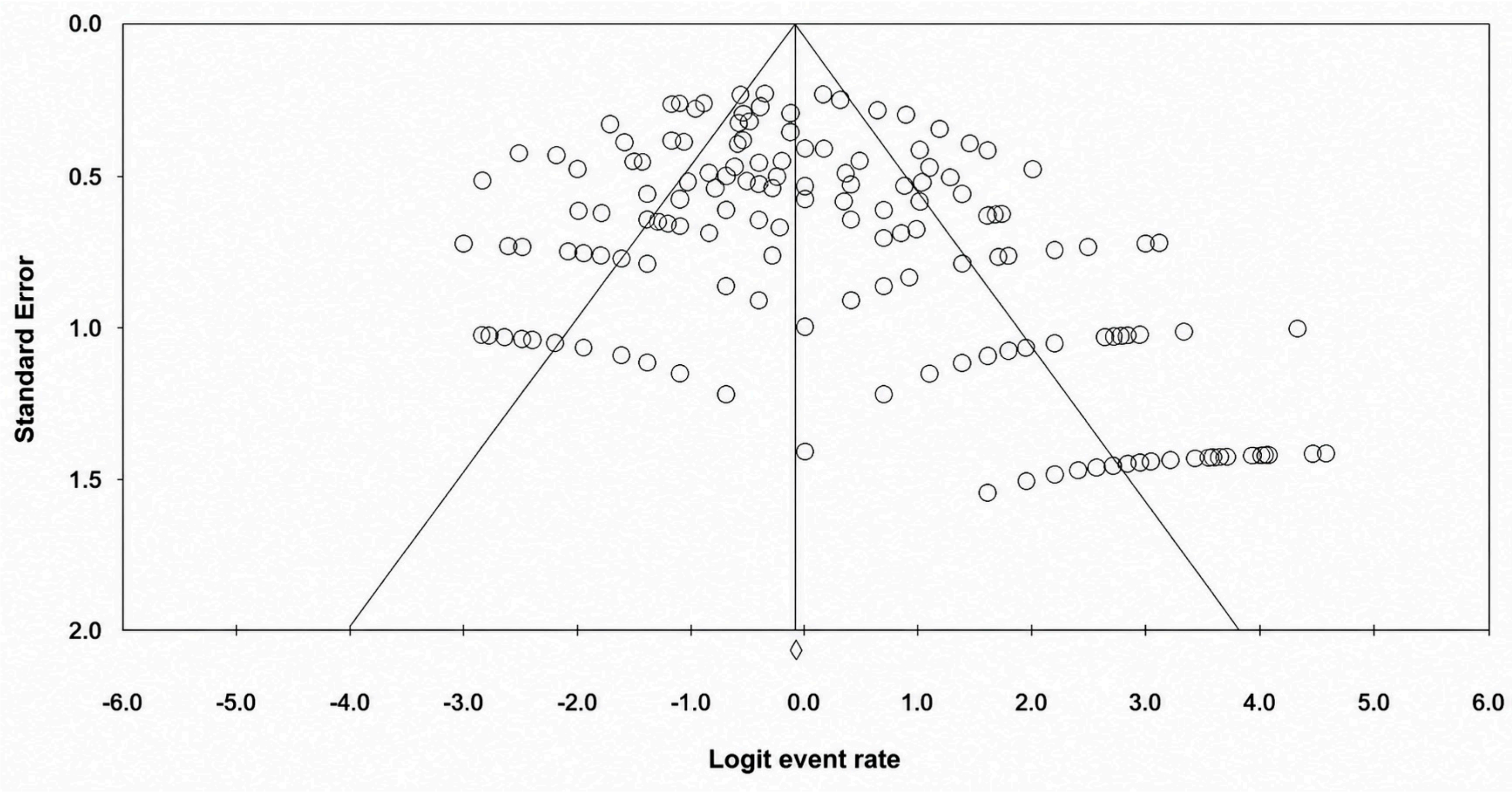

Figure S1. Funnel plot for assessment of publication bias.
