# Supplementary material for: Patterns of Anthelmintic Resistance in Gastrointestinal Nematodes of Small Ruminants: A Systematic Review and Meta-Analysis
Source: Vet Sci. 2026 Oct 12;13(10):1077. doi: 10.3390/vetsci13101077 (PMC13709475; doi:10.3390/vetsci13101077)
Supplement: Supplementary file 1 [file vetsci-13-01077-s001.zip › Table S2.pdf]

**Table S2** Search strategies used to identify studies reporting anthelmintic resistance in gastrointestinal nematodes of small ruminants

| **Database (search date )** | **Search strategy** | Filters applied/restrictions |
|---|---|---|
| PubMed (28/03/2026) | (“sheep” OR “goats” OR “small ruminants”) AND (“AHR” OR “anthelmintic resistance” OR “susceptibility” OR “efficacy”) AND (“GINs” OR “endoparasites” OR “*Nematodirus* spp.” OR “*Cooperia* spp.” OR “*Haemonchus* spp.” OR “*Trichostrongylus* spp.” OR “*Teladorsagia* spp.”) AND (“anthelmintic drugs” OR “benzimidazoles” OR “macrocyclic lactones” OR “imidazothiazoles” OR “salicylanilides” OR “tetrahydropyrimidines” OR “amino-acetonitrile derivatives” OR “organophosphates”) | Filters applied: Full text; publication date from 1996 to March 2026. |
| Web of Science (28/03/2026) | (“sheep” OR “goats” OR “small ruminants”) AND (“AHR” OR “anthelmintic resistance” OR “susceptibility” OR “efficacy”) AND (“GINs” OR “endoparasites” OR “*Nematodirus* spp.” OR “*Cooperia* spp.” OR “*Haemonchus* spp.” OR “*Trichostrongylus* spp.” OR “*Teladorsagia* spp.”) AND (“anthelmintic drugs” OR “benzimidazoles” OR “macrocyclic lactones” OR “imidazothiazoles” OR “salicylanilides” OR “tetrahydropyrimidines” OR “amino-acetonitrile derivatives” OR “organophosphates”) | Filters applied: Article; publication year from 1996 to March 2026. |
| ScienceDirect (28/03/2026) | (“sheep” OR “goats” ) AND (“anthelmintic resistance” ) AND (“GINs” OR “*Haemonchus* spp.”) AND (“anthelmintic drugs” OR “benzimidazole” ) | Filters applied: Research articles; publication year from 1996 to March 2026; open access. |
| Google scholar (29/03/2026) | (“sheep” OR “goats” OR “small ruminants”) AND (“AHR” OR “anthelmintic resistance” OR “susceptibility” OR “efficacy”) AND (“GINs” OR “endoparasites” OR “*Nematodirus* spp.” OR “*Cooperia* spp.” OR “*Haemonchus* spp.” OR “*Trichostrongylus* spp.” OR “*Teladorsagia* spp.”) AND (“anthelmintic drugs” OR | Filter applied: Publication date from 1996 to March 2026 |

| | | |
|---|---|---|
| | “benzimidazoles” OR “macrocyclic lactones” OR “imidazothiazoles” OR “salicylanilides” OR “tetrahydropyrimidines” OR “amino-acetonitrile derivatives” OR “organophosphates”) | |
