# Supplementary material for: Patterns of Anthelmintic Resistance in Gastrointestinal Nematodes of Small Ruminants: A Systematic Review and Meta-Analysis
Source: Vet Sci. 2026 Oct 12;13(10):1077. doi: 10.3390/vetsci13101077 (PMC13709475; doi:10.3390/vetsci13101077)
Supplement: Supplementary file 1 [file vetsci-13-01077-s001.zip › Table S3.pdf]

Table S3. List of studies on anthelmintic resistance (AHR) in gastrointestinal nematodes of small ruminants globally during the study period (1996–2026).

References	Continent/ Country	Anthelmintic drug class (GIN isolates or farms with AHR/ Total isolates or farms sampled)	GIN genera reported	Host species	mode of administration	Bioassay techniques
	**Africa**					
Wanyangu et al. [97]	Kenya	IMD (9/35); BZ (10/28)<br>IMD (12/24); BZ (6/20)	*Haemonchus* spp.	Sheep<br>Goat	NS	F
Mukaratirwa et al. [98]	Zimbabwe	BZ (4/5); BZ (2/3); BZ (1/1)	*Haemonchus* spp.	Sheep	NS	F
Boersema and Pandey [99]	Zimbabwe	BZ (7/10); SLD (6/10)<br>IMD (4/10)	*Haemonchus* spp.	Sheep	Oral<br>Subcutaneous	F
Van Wyk et al. [100]	South Africa	IMD(6/9); SLD (3/12); BZ (6/12); ML (2/6<br>BZ (7/12); ML (5/6)	*Haemonchus* spp.	Sheep<br>Goat	NS	F
Gabriel et al. [101]	Zambia	BZ (5/6); ML (2/6)	*Haemonchus* spp.	Sheep	Oral	F
Dreyer [102]	South Africa	BZ (9/15); IMD (6/15); ML (3/15)	NS	Sheep	Oral	F
Atanasio et al. [103]	Mozambique	BZ (2/12); BZ (2/12); IMD (4/12)	*Haemonchus* sp.; *Trichostrongylus* spp.; *Oeshagostomum* spp.	Goat	Oral	F
Keyyu et al. [104]	Tanzania	BZ (1/1)	*Haemonchus* spp.	Sheep	Oral	F
Bakunzi [105]	South Africa	SLD (3/10); IMD (2/10); BZ (1/10)	NS	Goat	Oral	F
Changa and Kassuku [106]	Tanzania	BZ (8/8); IMD+SLD (8/8); ML (8/8)	*Haemonchus* spp.; *Trichostrongylus* spp.	Sheep	NS	F

Bentounsi et al. [107]	Algeria	ML (3/14); BZ (12/14)	*Teladorsagia* spp.; *Trichostrongylu* spp.	Sheep	NS	F
Bakunzi [108]	South Africa	SLD (1/12); IMD (4/12); BZ (1/12)	NS	Sheep	Oral	
Mirambo and Kassuku [109]	Tanzania	BZ (1/2)	*Haemonchus* spp.	Goat	NS	F
		IMD (2/2); BZ (1/2); IMD (2/2)		Sheep		
Kumsa and Abebe [110]	Ethiopia	IMD (1/1); BZ (1/1)	*Haemonchus* spp.	Goat	Oral	F
		ML (1/1)			Subcutaneous	
Bakunzi et al. [111]	South Africa	SLD (4/10); ML (4/10); BZ (1/10)	NS	Sheep	Oral	F
		SLD (4/10); ML (6/10); BZ (4/10)		Goat		
Tsotetsi et al. [112]	South Africa	BZ (2/3); IMD (2/3)	*Haemonchus* spp.;*Trichostrongylus* spp.; *Teladorsagia* spp.	Sheep	Oral	F
		ML (2/2)		Goats	Subcutaneous	
		BZ (2/2); IMD (2/2)	*Haemonchus* spp.		Oral	
Nsereko et al. [113]	Uganda	BZ (3/3); IMD (3/3)	*Haemonchus* spp.	Goat	Oral	F
		ML (3/3)			Subcutaneous	
Nabukenya et al.[114]	Uganda	BZ (4/16); IMD (10/17)	*Haemonchus* spp.	Sheep	Oral	F
		ML (8/12)			Subcutaneous	
Bahiru et al. [115]	Ethiopia	ML (1/1)	NS	Sheep	Subcutaneous	F
Sailen et al. [116]	Tanzania	BZ (1/1); IMD (1/1)	*Haemonchus* spp.	Goat	Oral	F
		BZ (1/1); IMD (1/1)		Sheep		
Atanásio-Nhacumbe et al. [117]	Mozambique	BZ (8/11)	*Haemonchus* spp.	Goat	Oral	F

Atanásio-Nhacumbe et al. [118]	Mozambique	BZ (8/12)	*Haemonchus* spp.	Goat	Oral	F
Ramabu et al. [119]	Botswana	ML (2/10)	NS	Goat	Oral	F
Aboelhadid et al. [14]	Egypt	BZ (1/1)<br>BZ (1/1)	*Haemonchus* spp.	Sheep	Oral<br>-	F<br>ASP
Mphahlele et al. [34]	South Africa	BZ (4/5); IMD (3/5)<br>ML (3/5)<br>BZ (5/5)<br>BZ (2/5)	*Haemonchus* spp.<br>*Haemonchus* spp.<br>*Haemonchus* spp.<br>*Haemonchus* spp.	Sheep	Oral<br>Subcutaneous<br>-<br>-	F<br><br>E<br>M
Van der Merwe [8]	South Africa	OP (1/1); IMD+SLD (1/1); ML (1/1); IMD (1/1)	*Haemonchus* spp.	Sheep	Oral	F
Idika et al. [120]	Nigeria	ML (1/8)	*Trichostrongylus* spp.<br>*Haemonchus* spp.	Goat	NS	F
Onyeabor et al. [121]	Nigeria	ML (1/1); BZ (1/1); IMD (1/1)	NS	Goat	Oral	F
Wondimu and Bayu [122]	Ethiopia	BZ (1/1); SLD (1/1); IMD (1/1); ML (1/1)	*Trichostrongylus* spp.;<br>*Haemonchus* spp.;<br>*Oesophagostomum* spp.	Goat	Oral	F
Stephanie et al. [123]	Cameroon	ML (2/5)	*Trichostrongylus* spp.<br>*Haemonchus* spp.;<br>*Oesophagostomum* spp.	Shepp	NS	F
Emsley et al. [23]	South Africa	BZ (3/3); IMD (3/3)<br>ML (3/3)	*Haemonchus* spp.	Sheep	Oral<br>subcutaneous	F

		BZ (3/3)			-	E
		BZ (3/3)				L
Mushonga et al. [124]	Zimbabwe	ML (1/1); BZ (1/1); IMD (1/1)	*Haemonchus* spp.	Sheep	NS	F
Guinda et al. [125]	Mozambique	BZ (5/16)	*Haemonchus* spp.; *Trichostrongylus* spp	Goat	Oral	F
Ambaw et al. [126]	Ethiopia	IMD (3/3); BZ (3/3) ML (3/3)	*Haemonchus* spp.; *Trichostrongylus* spp.; *Teladorsagia* spp.; *Trichuris* spp.	Sheep	Oral subcutaneous	F
Mavundela et al. [2]	South Africa	BZ+SLD (6/6); IMD+ISN (1/1); ML (6/6); IMD (3/3)	*Haemonchus* spp.	Sheep	Oral	F
	**Asia**					
Saddiqi et al. [127]	Pakistan	BZ (2/2); BZ (2/2); BZ (2/2)	*Haemonchus* spp.; *Trichostrongylus* spp.	Sheep	Oral	F
Saeed et al. [128]	Pakistan	BZ (6/18)	*Haemonchus* spp.; *Trichostrongylus* spp.; *Cooperia* spp.; *Teladorsagia* spp; *Oesophagostomum* spp.	Goat	Oral	F
		BZ (6/18)			-	E
Köse et al. [129]	Turkey	ML (7/7)	*Haemonchus* spp.; *Oesophagostomum* spp.	Sheep	subcutaneous	F
Mohamed and Al-Farwachi [130]	Iraq	BZ (4/6)	*Strongyloides* spp.; *Marshallian* sp.; *Nematodirus* sp.; *Haemonchus* spp.	Sheep	Oral	F

Singh and Gupta [131]	India	ML (3/25)	*Haemonchus* spp.	Shepp	Subcutaneous	F
		THP (3/25)	*Haemonchus* spp.		Oral	
Saeed et al. [132]	Pakistan	IMD (15/18); BZ (6/18)	*Haemonchus* spp.; *Trichostrongylus* spp.; *Teladorsagia* sp.	Goat (Beetal)	Oral	F
Rialch et al. [133]	India	BZ (2/14)	*Haemonchus* spp.	Sheep	Oral	F
		BZ (6/14)		Goat		
		BZ (2/14)		Sheep		E
		BZ (6/14)		Goat	-	
		BZ (2/14)		Sheep		L
		BZ (6/14)		Goat		
Abubakar et al. [134]	Malaysia	BZ (2/2); IMD (2/2)<br>ML (2/2)	NS	Goat	Oral<br>Subcutaneous	F
Gelot et al. [135]	India	BZ (10/10); IMD (10/10)<br>ML (10/10)	*Haemonchus* spp.<br>*Trichostrongylus* spp.	Goat	Oral<br>Subcutaneous	F
Singh et al. [136]	India	BZ (2/2)	NS	Sheep	Oral	F
		BZ (2/2)		Goat		
Islam et al. [137]	Bangladesh	BZ (1/1)	*Haemonchus* spp.	Sheep	Oral	F
Dyary [138]	Iraq	ML (19/19); BZ (16/19); IMD (14/19)	*Nematodirus* spp.	Sheep	Oral	F
Pawar et al. [9]	India	BZ (3/3); ML (1/3); IMD (2/3)	*Haemonchus* spp.; *Trichostrongylus* spp.; *Oesophagostomum* spp.; *Chabertia* sp.; *Strongyloides* spp.	Goat	Oral	F

		BZ (2/2); ML (2/2); IMD (2/2)	*Haemonchus* spp.; *Trichostrongylus* spp.; *Oesophagostomum* spp.; *Chabertia* sp.; *Strongyloides* spp.	Sheep		
Muhammad et al. [139]	Pakistan	BZ (15/15); IMD (15/15); ML (15/15)<br>IMD+ML (15/15)	*Haemonchus* spp.	Sheep	Oral<br>Subcutaneous	F
Dyary and Banaz [17]	Iraq	BZ (3/3); ML (3/3); IMD (3/3)<br>ML (3/3)	*Trichostrongylus* spp.	Sheep	Oral<br>-	F<br>L
Ratanapob et al. [21]	Thailand	BZ (29/29)<br>ML (29/29); IMD (2/29)	NS	Goat	Oral<br>Subcutaneous	F
Rerkyusuke et al. [140]	Thailand	ML (5/6); ML(1/4)<br>ML (5/6)	*Haemonchus* spp.; *Trichuris* spp.	Goat	Subcutaneous<br>Pour-on	F
Kalkal and Vohra [141]	India	BZ (4/4)<br>BZ (3/4)	*Haemonchus* spp.	Goat	Oral<br>-	F<br>E
Nath et al. [142]	India	BZ (3/3)	*Haemonchus* spp.	Goat	Oral	F
Chan et al. [143]	Thailand	BZ (18/20)	*Haemonchus* spp.; *Trichostrongylus* sp.	Goat	-	E
		IMD (5/7)	*Haemonchus* spp.			L
		BZ (20/20)	*Haemonchus* spp.; *Trichostrongylus* sp.		-	ASP
		IMD (24/26)	*Haemonchus* spp.			
Mukbel et al. [144]	Jordan	BZ (6/6)<br>ML (6/6); IMD (4/6)	*Haemonchus* spp.	Sheep	Oral<br>Subcutaneous	F

Kalkal et al. [145]	India	BZ (4/4)	*Haemonchus* spp.; *Trichostrongylus* spp.; *Strongyloides* spp.	Sheep	Oral	F
		BZ (2/4)	*Haemonchus* spp.		-	E
Kaur et al. [146]	India	BZ (17/17); IMD (17/17); ML (16/17)	*Haemonchus* spp.; *Trichostrongylus* spp.	Goat	Oral	F
		BZ (12/17)	*Haemonchus* spp.; *Trichostrongylus* spp.		-	E
		IMD (15/16); ML (11/15)	*Haemonchus* spp.; *Trichostrongylus* spp.			L
Rompo et al. [147]	Thailand	BZ (9/9); BZ+ML (12/12) ML (9/9)	*Haemonchus* spp.; *Trichostrongylus* spp.	Goat	Oral Subcutaneous	F
	**Oceania**					
Waghorn et al. [148]	New Zealand	BZ (33/80); BZ+IMD (6/80)	*Ostertagia* spp.; *Cooperia* spp.; *Nematodirus* spp.;*Trichostrongylus* spp.; *Haemonchus* spp.	Sheep	NS	F
		IMD (19/80)	*Nematodirus* spp.; *Teladorsagia* spp.; *Trichostrongylus* spp.			
		ML (20/80); ML (29/80)	*Ostertagia* spp.; *Cooperia* spp.; *Nematodirus* spp.;*Trichostrongylus* spp.			

Hughes et al. [149]	New Zealand	ML (22/47); BZ (41/76); IMD (18/49); BZ+IMD (2/42)	*Teladorsagia* sp.	Sheep	Oral	F
Sutherland et al. [150]	New Zealand	ML (1/1); BZ+ IMD (1/1)	*Teladorsagia* spp., *Trichostrongylus* spp.; *Haemonchus* spp.	Sheep	NS	F
Lyndal-Murphy et al. [13]	Australia	BZ+IMD+ML+SLD (4/20); IMD (5/19); OP (1/13); SLD+ML (6/18); ML (7/16)	*Haemonchus* spp.	Sheep	Oral	F
Preston et al. [19]	Australia	ML (36/55);ML (18/65); ML (6/59); BZ + IMD (38/66); ML+BZ+IMD (1/6); OP+BZ+IMD (6/33); OP+ML (1/17)	*Trichostrongylus* spp; *Teladorsagia* spp.	Sheep	NS	F
Kour et al. [25]	Fiji	IMD (1/4); BZ (4/4); ML (1/4);ML (2/4)	*Haemonchus* spp.; *Trichostrongylus* spp.	Goats	Oral	F
		IMD (3/3); BZ (2/3); BZ+IMD (2/3); ML (1/3)		Sheep		
	**Europe**					
Mortensen et al. [151]	Georgia	BZ (14/15); ML (17/18); IMD (6/18); ML (1/18)	*Haemonchus* spp.	Goats	Oral	F
Traversa et al. [152]	Italy	IMD (3/3); ML (2/3)	*Teladorsagia* sp.; *Trichostrongylus* spp.	Sheep	NS	F
Díez-Baños et al. [153]	Spain	BZ (11/72)	*Teladorsagia* spp.; *Trichostrongylus* spp.;	Sheep	NS	F

			Nematodirus spp.; *Chabertia* sp.			
		ML (4/72)	*Teladorsagia* sp.			
		BZ (21/72)			-	E
Zanzani et al. [154]	Italy	BZ (3/15); ML (1/15)	*Teladorsagia* spp.	Goats	Oral	F
		BZ (3/15); ML (1/15)	*Trichostrongylus* spp.			
		ML (1/15)	*Haemonchus* spp.			
Holm et al. [155]	Denmark	ML (8/10)	*Haemonchus* spp.	Goats	Subcutaneous	F
		BZ (8/10)			Oral	
		BZ (8/10)			-	E
Murri et al. [156]	Switzerland	ML (43/43)	*Haemonchus* spp.; *Trichostrongylus* spp.; *Teladorsagia* spp.	Goat	Pour-on	F
Geurden et al. [157]	France	BZ (10/10); BZ (10/10)	*Trichostrongylus* spp.;	Sheep	Oral	F
	Greece	IMD (4/5); BZ (5/6)	*Teladorsagia* spp.;			
	Italy	IMD (2/4); BZ (1/2)	*Oesophagostomum* spp.			
Kupčinskas et al. [158]	Lithuania	BZ (3/15)	*Teladorsagia* spp.;	Sheep	Oral	F
		ML (2/16)	*Trichostrongylus* spp.		Subcutaneous	
Kupčinskas et al. [159]	Lithuania	BZ (25/25)	*Teladorsagia* spp.;	Sheep	-	E
		ML (13/21)	*Trichostrongylus* spp.			M
Devos et al. [160]	France	BZ (11/13)	NS	Goat	Oral	F
		ML (10/13);			Pour- on	
Milhes et al. [161]	France	BZ (3/4); ML (1/4)	*Trichostrongylus* spp.; *Teladorsagia* spp.	Sheep	Oral	F

Ploeger and Everts [162]	Netherlands	BZ (22/30); ML (18/23); ML (15/32); AAD (2/26)	*Haemonchus* spp.; *Teladorsagia* spp.; *Trichostrongylus* spp.	Sheep	NS	F
Lambertz et al. [163]	Italy	ML (27/27)	*Haemonchus* spp. *Teladorsagia* spp.; *Trichostrongylus* spp.	Sheep	Oral	F
		BZ+SLD (27/27)	*Haemonchus* sp.; *Trichostrongylus* spp.			
		BZ (27/27)	*Teladorsagia* spp.			
		IMD+SLD (27/27)	*Teladorsagia* spp.			
Mickiewicz et al. [164]	Poland	BZ (2/2)	*Haemonchus* spp.		Oral	F
		ML (2/2)	*Haemonchus* spp.		Subcutaneous	
		IMD (1/2)	*Trichostrongylus* spp.		Oral	
		BZ (2/2)	*Haemonchus* spp.	Goat		
		ML (2/2);IMD (1/2); BZ (2/2)	*Trichostrongylus* spp.; *Teladorsagia* spp.			L
		BZ (2/2)	*Teladorsagia* spp.; *Haemonchus* spp.		-	ASP
Claerebout et al. [165]	Belgium	BZ (8/8); ML (7/20)	*Haemonchus* spp.; *Teladorsagia* spp.	Sheep	NS	F
Potârniche et al. [166]	Romania	ML (10/10)	*Haemonchus* spp.	Goat	Pour-on	F
		BZ (10/10)			-	E
		ML (10/10); BZ (10/10)				L
Vadlejch et al. [18]	Czech Republic	BZ (18/24)<br>ML (13/24)	*Haemonchus* spp.; *Teladorsagia* spp.;	Goats	-	E<br>L

			Trichostrongylus spp.			
Mickiewicz et al. [167]	Poland	BZ (37/42); ML (40/42); IMD (5/42)	*Haemonchus* spp.; *Trichostrongylus* spp.	Goat	-	L
Keidāne et al. [168]	Latvia	BZ (4/10) ML (9/10) BZ+ML (1/3)	*Haemonchus* spp.	Sheep	NS	F
Höglund et al. [20]	Sweden	BZ (11/30); ML (36/47)	*Haemonchus* spp.	Sheep	Oral	F
Krücken et al. [24]	Germany	BZ (7/8); ML (8/12);	*Haemonchus* spp.; *Teladorsagia* spp.	Sheep	Oral	F
		ML (8/8)			Subcutaneous	
D'Ambrosio et al. [169]	Italy	BZ (2/18)	*Trichostrongylus* spp.; *Haemonchus* spp.	Sheep	Oral	F
	**Latin America**					
Thomaz-Soccol et al. [170]	Brazil	ML (34/42); ML (9/38); IMD (15/42); BZ (35/42); SLD (16/42); BZ+SLD (6/31)	NS	Sheep	NS	F
Klauck et al. [171]	Brazil	SLD (2/2); IMD (2/2); BZ (4/4); BZ+IMD (1/1)	*Haemonchus* spp.; *Trichostrongylus* spp.	Sheep	Oral	F
García et al. [172]	Colombia	BZ (2/3); BZ (3/4); ML (2/2); ML (1/1); IMD (2/2)	*Haemonchus* spp.	Sheep	NS	F
Flávia da Silva et al. [173]	Brazil	BZ (19/20); ML (17/20); SLD (16/20); IMD (8/20); AAD (9/20)	*Haemonchus* spp.; *Oesophagostomum* spp.; *Trichostrongylus* spp.	Sheep	Oral	F

Mallmann Júnior et al. [174]	Brazil	SLD (7/7); IMD (7/7); BZ (7/7); AAD (3/7)	*Haemonchus* spp. *Trichostrongylus* spp.;	Sheep	Oral	F
		ML (7/7)	*Oesophagostomum* spp.; *Teladorsagia* spp.		Subcutaneous	
Ramos et al. [15]	Brazil	ML (7/7); ML (7/7); ML (6/7); IMD (5/7); BZ (7/7)	*Haemonchus* spp.	Sheep	Subcutaneous	F
		SLD (7/7)			Oral	
		BZ (7/7)			-	ASP
Soares et al. [175]	Brazil	ML (2/2); IMD (2/2); BZ (2/2)	NS	Sheep	Oral	F
		ML (2/2); IMD (2/2); BZ (2/2)		Goats		
Batista et al. [22]	Brazil	BZ (12/12); ML (12/12); IMD (3/12)	*Haemonchus* spp.; *Trichostrongylus* spp.	Sheep	Oral	F
Lima Júnior et al. [176]	Brazil	IMD (1/1); SLD (2/2); OP (1/2); BZ (5/5)	NS	Sheep	Oral	F
		ML (4/4); ML (3/3); NST (3/3)			Subcutaneous	
	**North America**					
George et al. [177]	Trinidad	BZ (2/2); BZ (3/3); IMD (2/2)	NS	Sheep	Oral	F
Falzon et al. [178]	Canada	ML (28/29);BZ (19/20); IMD (1/17)	*Haemonchus* spp.	Sheep	Oral	F
		BZ (3/21); BZ (13/21)			-	L
Herrera-Manzanilla et al. [179]	Mexico	BZ (7/7); ML (7/7)	*Haemonchus* spp.; *Trichostongylus* spp.; *Oesophagostomum* spp.	Sheep	Subcutaneous	F

		IMD (7/7)	*Haemonchus* spp.; *Trichostongylus* spp.			
Alcalá-Canto et al. [180]	Mexico	BZ (3/3); SLD (3/3); BZ (3/3); BZ+SLD (3/3)	NS	Sheep	Oral	F
Santiago-Figueroa et al. [181]	Mexico	BZ (8/8)<br>ML (4/5); IMD (1/5)	*Haemonchus* spp.;*Trichostrongylus* spp.; *Cooperia* spp.; *Oesophagostomum* spp.	Sheep	Oral<br>Subcutaneous	F
		BZ (8/8)	*Haemonchus* spp.		-	E
		BZ (8/8)	*Haemonchus* spp.			ASP
Mondragón-Ancelmo et al. [182]	Mexico	BZ (28/28)<br>ML (29/29)	*Cooperia* spp.; *Trichostrongylus* spp.	Sheep	Oral<br>Subcutaneous	F
Sepúlveda-Vázquez et al. [183]	Mexico	BZ (9/9); BZ (7/8)<br>ML (9/9); ML (12/14); IMD (4/9); IMD (6/10)	*Haemonchus* spp.	Sheep	Oral<br>Subcutaneous	F
Queiroz et al. [26]	Canada	BZ (76/77)<br>BZ (23/57)<br>BZ (39/55)	*Haemonchus* spp.<br>*Teladorsagia* spp.<br>*Trichostrongylus* spp.	Sheep	-	DSP
	**Central America**					
Castro-Arnáez et al. [16]	Costa Rica	BZ (48/48)	*Haemonchus* spp.; *Trichostrongylus*	Sheep	Subcutaneous	F

		spp.;*Oesophagostomum* spp.; *Teladorsagia* spp.
	ML (45/47)	*Haemonchus* spp.; *Trichostrongylus* sp.; Oesophagostomum spp.; *Teladorsagia* sp.; *Chabertia* sp.; *Cooperia* sp.
	IMD (8/47)	*Haemonchus* spp.; *Trichostrongylus* sp.

Abbreviations: NS, not specified; AAD, amino-acetonitrile derivative; NTS, nitrophenol substitutes; THP, Tetrahydropyrimidine; OP, organophosphate; SLD, Salicylanilides; BZ + IMD, coformulation of Benzimidazoles + Imidazothiazoles; IMD + SLD, coformulation of Imidazothiazoles + Salicylanilides; BZ + SLD, coformulation of Benzimidazoles + Salicylanilides; BZ + ML, coformulation of Benzimidazoles + Macrocyclic lactones; IMD + ISN, coformulation of Imidazothiazoles + Isoquinoline derivatives; IMD + ML, coformulation of Imidazothiazoles + Macrocyclic lactones; BZ + IMD + ML + SLD, coformulation of Benzimidazoles + Imidazothiazoles + Macrocyclic lactones + Salicylanilides; SLD + ML, coformulation of Salicylanilides + Macrocyclic lactones; ML + BZ + IMD, coformulation of Macrocyclic lactones + Benzimidazoles + Imidazothiazoles; OP + BZ + IMD, coformulation of Organophosphates + Benzimidazoles + Imidazothiazoles; OP + ML, coformulation of Organophosphates + Macrocyclic lactones; GIN, gastrointestinal nematodes; TBZ, thiabendazole; DD, discrimination dose

Criteria for quantifying anthelmintic resistance

F – FECRT (faecal egg count reduction test): AHR was defined when the FECR was < 95% and the 95% confidence interval was < 90%; E – EHT (egg hatch test): AHR was defined as ≥ 99% egg hatching at the DD of 0.1 μg/mL TBZ; L – LDT (larval development test): AHR was defined as ≥ 50% survival of L3 larvae at the DD of 0.1 μg/mL TBZ; M – MALDT (micro-agar larval development test): AHR was defined based on the proportion of larvae developing from L1 to L3 at the DD of 0.02 μg/mL; ASP – Allele-Specific PCR: AHR was assessed by detecting benzimidazole resistance-associated alleles in the isotype-1 β-tubulin gene, identified by amplification of diagnostic 200/250 base pair fragments.; DSP – Deep Amplicon Sequencing PCR: AHR was assessed by sequencing the isotype-1 β-tubulin gene and quantifying the frequency of benzimidazole resistance-associated mutations at codons F167Y, E198L, and F200Y.
